# A Secure Scheme for Distributed Consensus Estimation against Data Falsification in Heterogeneous Wireless Sensor Networks

**DOI:** 10.3390/s16020252

**Published:** 2016-02-19

**Authors:** Shichao Mi, Hui Han, Cailian Chen, Jian Yan, Xinping Guan

**Affiliations:** 1 Luoyang Electronic Equipment Test Center (LEETC), Luoyang 471003, China; cemee_hanhui@163.com; 2 Department of Automation, Shanghai Jiao Tong University, and Key Laboratory of System Control and Information Processing, Ministry of Education of China, Shanghai 200240, China; cailianchen@sjtu.edu.cn (C.C.); jyan@ysu.edu.cn (J.Y.); xpguan@sjtu.edu.cn (X.G.)

**Keywords:** security, relay nodes, data falsification, distributed estimation, heterogeneous wireless sensor networks

## Abstract

Heterogeneous wireless sensor networks (HWSNs) can achieve more tasks and prolong the network lifetime. However, they are vulnerable to attacks from the environment or malicious nodes. This paper is concerned with the issues of a consensus secure scheme in HWSNs consisting of two types of sensor nodes. Sensor nodes (SNs) have more computation power, while relay nodes (RNs) with low power can only transmit information for sensor nodes. To address the security issues of distributed estimation in HWSNs, we apply the heterogeneity of responsibilities between the two types of sensors and then propose a parameter adjusted-based consensus scheme (PACS) to mitigate the effect of the malicious node. Finally, the convergence property is proven to be guaranteed, and the simulation results validate the effectiveness and efficiency of PACS.

## 1. Introduction

With the features of being low cost, easy to deploy and the self-organization of sensor nodes, wireless sensor networks (WSNs) are widely used to estimate the physical parameters in hazardous and remote areas, such as military defense, intelligent transportation, industrial production, environment monitoring, smart homes, and so on [1,2,3,4,5]. In most applications, the environment may be complicated and needs different kinds of sensors. Therefore, it is wiser to use a combination of different sensors: numbers of cheap, low-end sensors and some expensive, high-quality sensors, making up the heterogeneous wireless sensor networks (HWSNs) [6,7,8,9].

In WSNs, it is often necessary for some or all of the nodes to calculate some functions with certain parameters. Additionally, distributed estimation is an effective tool to exchange information among sensor nodes in the network. It is a well-studied domain and has attracted much attention [10,11,12,13,14,15,16]. However, in most applications, the networks are deployed in a harsh environment or a hostile region. Due to the restrictions of the computation abilities, storage capacity and battery power of sensor nodes, they are unable to be loaded with firewall-like security tools [17]. There inevitably exists a security problem in distributed networks. For example, if a sensor is intruded in an HWSN, other kinds of sensors are affected, and this may cause network congestion, network lifetime reduction, sensing inaccuracy, *etc.* [18,19,20]. Therefore, the study of the security of distributed estimation in HWSNs is very important for addressing the issue of the growing malicious attack threat.

As we know, distributed estimation in the presence of malicious nodes has attracted considerable attention in homogeneous wireless sensor networks [21,22,23]. Generally, fault monitoring algorithms are based on a detection threshold or state estimate residuals to distinguish attackers from honest nodes. In an HWSN, with different types of sensors, data are integrated in different ways. Therefore, the detection algorithms in homogeneous wireless sensor networks are not suitable for HWSNs. Nevertheless, some security schemes have been proposed for HWSNs. Incorporating pairwise keys used for sensor nodes communicating with each other has been studied in [24]. Key management schemes for providing security operations in the HWSN have been considered [25]. Some researchers were concerned with mutual authentication frameworks [26]. These secure schemes based on authentication and key management cost much energy and storage capacity, while sensor nodes have the restrictions of computation abilities, storage capacity and battery power. The schemes were not practical for low-cost sensors. Some secure schemes based on a distributed consensus estimation algorithm have also been proposed for wireless sensor networks. A weighted averaging-based consensus scheme (WACS) [27] was proposed to mitigate the negative impact of malicious nodes for homogeneous wireless sensor networks. The scheme was based on weighted average parameters, which were prescribed as fixed values within a certain range, and the parameters affected the convergence speed or there was the effect of the weighted averaging-based consensus scheme (WACS). The results with the WACS finally converged to the average of the initial values of all sensor nodes. If the initial values were forged by attackers, the scheme could do nothing to protect the network from attackers injecting false data into the sensing stage. Considering the restrictions of the computation abilities, storage capacity, the battery power of sensor nodes and the security of the network, this paper focuses on security issues in HWSNs.

In this paper, we consider distributed estimation in the HWSNs consisting of two types of sensors: sensor nodes (SNs) and relay nodes (RNs). SNs have a good hardware architecture and are high quality, and they can sense the surrounding parameters and are responsible for data fusion. RNs are inexpensive and low end, and their main role is to relay the information for SNs. In the case the network is attacked by malicious nodes, we present a data falsification attack, that is malicious nodes manipulate false data in the network and damage the consensus of the whole system. Then, we propose a parameter adjusted-based consensus scheme (PACS) to decrease the negative effect of the data falsification attack. The main difference from the existing algorithm is that we explicitly consider the heterogeneity of responsibilities between the two types of sensors. With the network topology, we demonstrate the transformation of the distributed consensus method to overcome the challenges produced by the heterogeneity. Additionally, by adjusting the weights of sensor nodes, we illustrate how the PACS can decrease the effect of malicious nodes and avoid excluding the honest nodes with large deviations to participate in the distributed consensus. We evaluate the effectiveness and efficiency of the PACS.

The reminder of the paper is outlined as follows. The network model and the attack model are introduced in Section 2. In Section 3, we propose the secure scheme. Section 4 provides some simulations. The conclusion and future work are presented in Section 5.

## 2. System Model

### 2.1. Network Model

For the large-scale network, the long distance transmission costs much energy and may result in separate groups of sensors. A popular method is to deploy relay sensors for connecting the separate groups. Thus, the whole network can be connected. Here, we consider a connected HWSN with a combination of different sensors: sensor nodes (SNs) and relay nodes (RNs). The network contains *N* nodes, which consists of *M* numbers of SNs and (N-M) numbers of RNs. $I_{S}=\left\{1,2,...,M\right\}$ represents the set of SNs, and $I_{R}=\left\{M+1,M+2,...,N\right\}$ represents the set of RNs. Each SN can perceive the surrounding parameters, while RNs cannot sense the parameters, but can relay information for the network. We use an undirected graph G=(V,E) to describe the network. V denotes the set of nodes, and $V=I_{S}\cup I_{R}$, where $I_{S}$ represents the set of SNs and $I_{R}$ represents the set of RNs; E∈V×V denotes the set of edges referring to the communication links. If there exists an edge connecting two nodes, the two nodes can communicate with each other. If (i,j)∈E where i≠j (*i.e.*, node *j* can transfer information with node *i*), we call node *j* a neighbor of node *i*. $N_{i}=\left\{j|\left(j,i\right)\in E\right\}\subset V$ represents the neighbor set of node *i*. The number of elements in $N_{i}$ is denoted by $|N_{i}|$.

Define the Laplacian matrix of G as $L={\left(l_{ij}\right)}_{N\times N}$, then:

(1) $$l_{ij}=\left\{\begin{array}{cc} -1, & \mathrm{if}\;j\neq i,j\in N_{i}\\ |N_{i}|, & \mathrm{if}\;j=i\\ 0, & \mathrm{otherwise} \end{array}\right.$$

Each node $i\in I_{S}$ is supposed to begin with a private value $x_{i}\left(0\right)$ by sensing the environment. The aim for the network is to converge to a common value relying on $x_{i}\left(0\right)$ by the incorporation of each node. During the distributed consensus estimation, at each iteration step *k*, each sensor node updates and exchanges its values with neighbors according to a prescribed strategy, which can be modeled by a discrete-time equation, and each node *i* updates its estimation as follows:

(2) $$x_{i}\left(k+1\right)=x_{i}\left(k\right)+\epsilon \sum_{j\in N_{i}}a_{ij}\left(x_{j}\left(k\right)-x_{i}\left(k\right)\right),\;\mathrm{if}\;i\in I_{S}$$

and:

(3) $$x_{i}\left(k+1\right)=\sum_{j\in N_{i}}\gamma_{ij}x_{j}\left(k\right),\;\mathrm{if}\;i\in I_{R}$$

where:

(4) $$0<\epsilon <(\max_{i}|N_{i}{\left|\right)}^{-1}=\frac{1}{\Delta }$$

$x_{i}\left(k\right)$ represents the state value of node *i* at time step *k*. Δ represents the maximum degree of the network, and $a_{ij}$ denotes the amplitude of the signal received by sensor *i* from sensor *j*. $\gamma_{ij}$ is the corresponding weight satisfying $\gamma_{ij}>0$, and $\sum_{j\in N_{i}}\gamma_{ij}=1,\forall i\in I_{R}$. We set the notation $\gamma_{ij}=0$, for $j\notin N_{i}$. For all $j\in N_{i}$, we have that $\sum_{j=1}^{N}\gamma_{ij}=1,\forall i\in I_{R}$. It is clear that each node updates its estimates by the linear combination of its neighbors’ state values and its own values.

To simplify the consensus scheme, we combine Equations (2) and (3) and get the consensus equation that only contains the SNs, but implies the state of RNs.

(5) $$x_{i}\left(k+1\right)=x_{i}\left(k\right)+\epsilon \left[\sum_{j\in N_{i}^{S}}a_{ij}\left(x_{j}\left(k\right)-x_{i}\left(k\right)\right)+\sum_{k\in N_{i}^{R}}\sum_{j\in N_{k}{}^{S}}a_{ik}\gamma_{kj}\left(x_{j}\left(k\right)-x_{i}\left(k\right)\right)\right]$$

$N_{i}^{S}$ represents the sensor neighbor set of node *i*, while $N_{i}^{R}$ represents the relay neighbor set of node *i*.

### 2.2. Attack Model

A familiar attack called data falsification is considered in this paper. A false state value may be manipulated in the sensing stage or in the state updating progress by a data falsification attacker. We can see that the data falsification attack is easy to implement if a sensor node has been captured. Moreover, due to noise in the environment, there is usually a large error when a sensor node perceives parameters. Therefore, it is hard to distinguish whether a sensor node is captured by data falsification or not. Since this kind of attack can effect the consensus process and cause long-term impacts, it can be destructive to the network. Three types of data falsification will be presented in the following [28].

*Perception Data Falsification (PDF) Attack*: This attack changes the value of $x_{i}\left(0\right),i\in I_{S}$. An attacker aims to forge a false sensing data and to disseminate it to its neighbors. However, in the information fusing phase, malicious nodes correctly update their estimates and send the estimated value to their neighbors. This kind of attack is easy to implement, but difficult to distinguish from honest nodes with a large deviation. To avoid mistaking honest nodes with a large deviation, the objective of our scheme in this paper is to decrease, but not to eliminate the attackers.

*Iteration Data Falsification (IDF) Attack*: False data are injected both in the sensing stage and at each iteration step by attackers. This type of attack can impact the consensus process; therefore, it can compromise the network for a long time.

*Random Data Falsification (RDF) Attack*: The attacker injects forged data or correctly executes a distributed estimation process in a random way. This type of attack is difficult to be detected because of its concealed feature.

In this paper, a distributed secure scheme based on parameter adjustment is presented to decrease the effect of the data falsification attack. We adjust the parameters in the distributed consensus algorithm (Equation (5) in Section 2.1). Abnormal nodes are distinguished from honest nodes via an adaptive local threshold. If a node is considered to be abnormal, the weight is reduced. In this way, we propose the PACS to decrease the effect of malicious nodes and to ensure the security of the network.

## 3. Secure Scheme

In this section, we propose a PACS for protecting the network from the data falsification attack. Then, its effectiveness is demonstrated by analyzing the algorithm.

### 3.1. Parameter Adjusted-Based Consensus Scheme

Detection algorithms are designed to assort abnormal nodes and honest nodes in the network. With the characteristics of the consensus algorithm, the state values of all of the nodes in the network converge to a common value, and the difference among all of the states is reduced to zero. Based on this characteristic, this paper presents a detection algorithm by comparing a localized threshold to the difference produced by each node state and its neighbors’, and the threshold adaptively shrinks to zero.

We now elaborate the consensus secure scheme based on the detection algorithm below. In the first stage, the node $i\in I_{S}$ makes a measurement independently and transfers the measurement value to its neighbors. Then, node *i* compares the state value to its neighbor’s value. The set of nodes satisfying $|x_{j}\left(k\right)-x_{i}\left(k\right)|<\lambda_{i}\left(k\right)$ is denoted as $N_{i}^{T}$, and the set of nodes satisfying $|x_{j}\left(k\right)-x_{i}\left(k\right)|\geq \lambda_{i}\left(k\right)$ is denoted as $N_{i}^{F}$. $\alpha_{i}\left(k\right)$ and $\beta_{i}\left(k\right)$ represent the number of $N_{i}^{F}$ and $N_{i}^{T}$, respectively. If $\beta_{i}\left(k\right)+1\geq \alpha_{i}\left(k\right)$, the measurement of the node $i\in I_{S}$ is correct, and its state update equation is demonstrated as follows.

(6) $$\begin{array}{ccc} & & x_{i}\left(k+1\right)=x_{i}\left(k\right)+\sigma \left(k\right)\left[\sum_{j\in N_{i}^{ST}}a_{ij}\left(x_{j}\left(k\right)-x_{i}\left(k\right)\right)+\sum_{j\in N_{i}^{SF}}\frac{a_{ij}}{a(k)}\left(x_{j}\left(k\right)-x_{i}\left(k\right)\right)\right.\\ & & +\sum_{k\in N_{i}^{R}}\sum_{j\in N_{k}{}^{ST}}a_{ik}\gamma_{kj}\left(x_{j}\left(k\right)-x_{i}\left(k\right)\right)+\left.\sum_{k\in N_{i}^{R}}\sum_{j\in N_{k}{}^{SF}}\frac{a_{ik}}{a(k)}\gamma_{kj}\left(x_{j}\left(k\right)-x_{i}\left(k\right)\right)\right] \end{array}$$

σ(k)>0 represents the weight. $N_{i}^{ST}$ denotes the set of sensor nodes in $N_{i}^{T}$, and $N_{i}^{SF}$ denotes the set of sensor nodes in $N_{i}^{F}$. a(k) is a parameter that can affect the consistency coefficient. From the equation, we can see that if a(k) becomes larger, the effect of the corresponding node becomes smaller. If a node is detected to be abnormal at the iteration step *k*, the corresponding coefficient a(k) becomes larger, and finally, its influence can be reduced. Additionally, a(k+1)=a×a(k), where *a* is an integer whose value is larger than one. If a node stops injecting false data at the iteration step, the corresponding coefficient a(k) decreases. If a(k)>1, a(k+1)=(1/a)×a(k) at the iteration step until a(k)=1. If a(k)=1 and the node performs normally, a(k) stays at one.

We define P(k)=I-σ(k)L. Then, we can get the compact form of Equation (6):

(7) x(k+1)=P(k)x(k)

Meanwhile, if the nodes satisfy $\beta_{i}\left(k\right)+1<\alpha_{i}\left(k\right)$, the nodes are considered to be incorrect, and their update equation remain as Equation (5).

To determine the threshold $\lambda_{i}\left(k\right)$ of each node $n_{i}$, we give the equation as follows.

(8) $$\lambda_{i}\left(k\right)=\frac{1}{|N_{i}|}\sum_{j\in N_{i}}\left|x_{j}\left(k\right)-\frac{x_{i}\left(k\right)+\sum_{j\in N_{i}}x_{j}\left(k\right)}{\left|N_{i}\right|+1}\right|$$

Considering that malicious nodes inject false data with a large deviation from the sensing data of authentic nodes, we can detect the attacker by comparing the threshold with the difference between the neighbors’ states and the state of node *i*. Furthermore, as the consensus is carried out, the localized threshold $\lambda_{i}\left(k\right)$ will decease to zero, and the attackers are given no tolerance.

The whole procedure of PACS is concluded in the following Algorithm 1.

**Algorithm 1:** Parameter Adjusted-Based Consensus Scheme (PACS).
**Require:** Graph G=(V,E), *M* SNs, N-M RNs
**Ensure:**
x(k)
1:set k=0
2:**for**
$i\in I_{S}$
**do**
3: set a(k)=1, a>1, $\alpha_{i}=0$ and $\beta_{i}=0$
4: *i* makes the measurement and gets the initial $x_{i}\left(0\right)$, then transmits $x_{i}\left(0\right)$ to its neighbors.
5:**end for**
6:**for** the consensus is not reached **do**
7: **for**
$i\in I_{S}$
**do**
8:  $\lambda_{i}\left(k\right)=\frac{1}{|N_{i}|}\sum_{j\in N_{i}}\left|x_{j}\left(k\right)-\frac{x_{i}\left(k\right)+\sum_{j\in N_{i}}x_{j}\left(k\right)}{\left|N_{i}\right|+1}\right|$
9:  **for**
$j\in N_{i}$
**do**
10:   **if**
$|x_{i}\left(k\right)-x_{j}\left(k\right)|>\lambda_{i}\left(k\right)$
**then**
11:    $\alpha_{i}=\alpha_{i}+1$
12:    **if**
a(k)=1
**then**
13:     a(k)=a
14:    **else**
15:     a(k)=a×a(k)
16:    **end**
**if**
17:   **else**
18:    $\beta_{i}=\beta_{i}+1$
19:    **if**
a(k)>1
**then**
20:     $a\left(k\right)=\frac{1}{a}a\left(k\right)$
21:    **else**
22:     a(k)=1
23:    **end**
**if**
24:   **end**
**if**
25:  **end**
**for**
26:  **if**
$\beta_{i}+1>\alpha_{i}$
**then**
27:   $x_{i}\left(k\right)$ updates its state according to Equation (6) and transmits the state estimation to its neighbors.
28:   Update ${\hat{L}}_{ii}$ and ensure the sum of *i*-th row sum is 1.
29:  **end**
**if**
30: **end**
**for**
31: set k=k+1
32:**end**
**for**

### 3.2. Performance Analysis

In this section, we analyze the performance of the proposed PACS. We consider that node $v_{1}$ is an abnormal node injecting false data in the sensing stage. The neighbors of $v_{1}$ are denoted by $v_{2},v_{3},...,v_{|N_{1}|+1}$. Then, at the iteration step *k*, we can get the Laplacian matrix L(k) as follows:

(9) $$l_{ij}=\left\{\begin{array}{cc} -\frac{a_{ij}}{a^{k}}, & \mathrm{if}\;j\neq 1,i\in N_{1}^{S}\\ -\frac{a_{ij}}{a^{k}}\gamma_{kj}, & \mathrm{if}\;j\neq 1,i\in N_{1}^{R}\\ l_{ij}-a_{ij}+\frac{a_{ij}}{a^{k}}, & \mathrm{if}\;j=i\neq 1,j\in N_{1}^{S}\\ l_{ij}-a_{ij}\gamma_{ij}+\frac{a_{ij}}{a^{k}}\gamma_{ij}, & \mathrm{if}\;j=i\neq 1,j\in N_{1}^{R}\\ l_{ij}, & \mathrm{otherwise} \end{array}\right.$$

According to the proposed algorithm, if the state value is considered abnormal at each iteration *k*, the corresponding parameter of $x_{1}\left(k\right)$ decreases. Additionally, P(k) is also changed. We suppose that the HWSN is not dominated by attackers.

Firstly, we illustrate that the network can reach convergence. Note that there are *n* eigenvalues of L(k) at the first place. According to the Gershgorin circle theorem [29] and $\gamma_{ij}<1$, we get the following inequations:

(10) $$|\xi_{m}-|N_{j}|+1-\frac{a_{ij}}{a^{k}}\gamma_{ij}\left|\leq \right|N_{j}|-1+\frac{a_{ij}}{a^{k}}\gamma_{ij},\forall j\in N_{1}^{R}$$

(11) $$|\xi_{m}-|N_{j}|+1-\frac{a_{ij}}{a^{k}}\left|\leq \right|N_{j}|-1+\frac{a_{ij}}{a^{k}},\forall j\in N_{1}^{S}$$

(12) $$|\xi_{m}-|N_{j}\left||\leq \right|N_{j}|,\forall j\notin N_{1}$$

where $\xi_{m}$ (1≤m≤n) is an eigenvalue of L(k) at the first place. Because of $0<\sigma \left(k\right)<{\left(\max_{i}N_{i}\right)}^{-1}$, we can get that $0\leq \xi_{m}\leq 2{\left(\max_{i}N_{i}\right)}^{-1}$. The eigenvalue of P(k) is $\xi_{m}^{*}=1-\sigma \left(k\right)\xi_{m}$, so we can obtain that $-1<\xi_{m}^{*}<1$. Moreover, the network is connected, and rank(G)=n-1 [4]. Thus, *L* has only a single zero eigenvalue, and P(k) has only one single eigenvalue, which is 1. The network can reach convergence.

With the consensus-based estimation, $x_{1}\left(k\right)$ finally converges to a normal range, and P(k) keeps a common value $P_{0}$ when *k* is large enough. We assume P(k) keeps a common value $P_{0}$ for $k=k_{0}$. Thus, we can get the result of the consensus.

(13) $$x\left(k\right)=\lim_{k\to \infty }P_{0}^{k}\prod_{k=1}^{k_{0}-1}P\left(k\right)x\left(0\right)$$

Secondly, we prove the efficiency of the scheme by computing $\lim_{k\to \infty }P_{0}^{k}\prod_{k=1}^{k_{0}-1}P\left(k\right)$. A lemma is introduced as follows.

**Lemma 1.**  *[13] Given a primitive nonnegative matrix* $P_{0}$*, if there exist eigenvectors u and* $w^{T}$ *satisfying* $P_{0}u=u$ *and* $w^{T}P_{0}=w^{T}$*, then* $\lim_{k\to \infty }P_{0}^{k}=\frac{uw^{T}}{w^{T}u}$ *holds*.

If the graph G is a strongly connected component, we can get that $P_{0}$ is a primitive nonnegative matrix [13]. The eigenvectors u=1 and $w^{T}=\left[1,a^{k},...a^{k}\right]$ satisfy the conditions in Lemma 1. Thus, we can obtain the following equation:

(14) $$\lim_{k\to \infty }P_{0}^{k}=\frac{uw^{T}}{1+a^{k}\left(N-1\right)}$$

According to the result of $\lim_{k\to \infty }P_{0}^{k}$, we can compute x(k). For the convenience of discussion, a two-hop network is considered. A theorem is presented as below. Additionally, a row vector $z_{i}^{T}$ is defined as follows:

(15) $$z_{i}^{T}=\left\{\begin{array}{cc} z_{1}, & \mathrm{if}\;i=1\\ z_{2}, & \mathrm{if}\;1<i\leq 1+|N_{1}|\\ z_{3}, & \mathrm{if}\;1+|N_{1}|<i\leq N \end{array}\right.$$

where $z_{3}\geq z_{2}\geq a^{k}z_{1}$. If a network has more hops, we can extend $z^{T}=\left[z_{1},z_{2},...,z_{2},z_{3},...,z_{3},...,z_{n},...,z_{N}\right]$ satisfying $z_{N}\geq ...\geq z_{3}\geq z_{2}\geq a^{k}z_{1}$, and a similar conclusion can be made. Define that $N_{i}^{G}=\{j|j\neq 1\;\mathrm{and}\;j\in N_{i}/N_{1},\;\forall 1<i\leq |N_{1}\left|+1\right\}$ and $N_{i}^{H}=\{j|j\in N_{i}⋂N_{1},\;\forall |N_{1}\left|+1<i\leq N\right\}$. The numbers of the above set are denoted as $|N_{i}^{G}|$ and $|N_{i}^{H}|$, respectively. The element number of the two-hop node set in the neighboring set of one-hop nodes is constant. Meanwhile, the element number of the one-hop node set in the neighboring set of two-hop nodes is constant, too. Thus, we get $|N_{2}^{G}\left|=\right|N_{i}^{G}|$; $|N_{3}^{H}\left|=\right|N_{i}^{H}|$ is invariable. Additionally, the following theorem is drawn.

**Theorem 1.**  *Given a row vector* $z^{T}$ *satisfying* $z_{3}\geq z_{2}\geq a^{k}z_{1}$ *and* $\psi^{T}=z^{T}P\left(k\right)$ *satisfying:*

(16) $$\psi_{i}^{T}=\left\{\begin{array}{cc} \psi_{1}, & if\;i=1\\ \psi_{2}, & if\;1<i\leq 1+|N_{1}|\\ \psi_{3}, & if\;1+|N_{1}|<i\leq N \end{array}\right.$$

*then* $\psi_{3}\geq \psi_{2}\geq a^{k}\psi_{1}$ *and* $\psi^{T}1=z^{T}1$ *as long as* $\sigma \left(k\right)\leq \frac{1}{|N_{2}^{G}\left|+\right|N_{3}^{H}|}$ *and* $\sigma \left(k\right)\leq \frac{1}{|N_{1}|+1}$.

**Proof.**  Since $\psi^{T}=z^{T}P\left(k\right)$, for all $j\in N_{1}^{S}$, we can get the following equation:

(17) $$\psi_{1}=z_{1}-\sigma \left(k\right)a_{1j}z_{1}|N_{1}|+\frac{a_{1j}}{a^{k}}\sigma \left(k\right)\left|N_{1}\right|z_{2}$$

and:

(18) $$\psi_{j}=\psi_{2}=\sigma \left(k\right)z_{1}+z_{2}-\frac{a_{1j}}{a^{k}}\sigma \left(k\right)z_{2}+\sigma \left(k\right)\left|N_{2}^{G}\right|\left(z_{3}-z_{2}\right),\forall j\in N_{i}^{G}$$

(19) $$\psi_{j}=\psi_{3}=z_{3}+\sigma \left(k\right)\left|N_{2}^{H}\right|\left(z_{2}-z_{3}\right),\forall j\in N_{i}^{H}$$

From the above equations, we have that:

(20) $$\psi^{T}1=\psi_{1}+|N_{1}|\psi_{2}+(N-1-|N_{1}\left|\right)\psi_{3}=z_{1}+|N_{1}|z_{2}+(N-1-|N_{1}\left|\right)z_{3}=z^{T}1$$

For all $j\in N_{1}^{R}$, the corresponding equations similar to the above Equations (17)–(20) are holds. Since the matrix P(k) eliminating the first column and the first row is a symmetric matrix, we get $|N_{1}\left|\right|N_{2}^{G}\left|=(N-1-\right|N_{1}\left|)\right|N_{3}^{H}|$.

Then, we compare $a^{k}\psi_{1}$, $\psi_{2}$ and $\psi_{3}$; for all $j\in N_{1}^{S}$.

(21) $$\psi_{2}-a^{k}\psi_{1}=\left(z_{2}-a^{k}z_{1}\right)\left[1-\sigma \left(k\right)\right](\frac{a_{1j}}{a^{k}}+|N_{1}\left|\right)+\sigma \left(k\right)\left|N_{2}^{G}\right|\left(z_{3}-z_{2}\right)$$

Since $\sigma \left(k\right)\leq \frac{1}{|N_{1}|+1}$, $\sigma \left(k\right)\leq \frac{1}{|N_{1}|+a^{-k}}$. Additionally, $z_{3}\geq z_{2}\geq a^{k}z_{1}$, we can get that $\psi_{2}-a^{k}\psi_{1}\geq 0$.

(22) $$\psi_{3}-\psi_{2}=\left(z_{3}-z_{2}\right)[1-\sigma \left(k\right)\left(\right|N_{3}^{H}\left|-\right|N_{2}^{G}\left|)\right]+\sigma \left(k\right)\left(\frac{a_{1j}}{a^{k}}z_{2}-z_{1}\right)$$

Because of $\sigma \left(k\right)\leq \frac{1}{|N_{2}^{G}\left|+\right|N_{3}^{H}|}$ and $z_{3}\geq z_{2}\geq a^{k}z_{1}$, the inequation $\psi_{3}-\psi_{2}\geq 0$ holds. When $j\in N_{1}^{R}$, the calculating process is similar; the inequations $\psi_{2}-a^{k}\psi_{1}\geq 0$ and $\psi_{3}-\psi_{2}\geq 0$ hold, too. ☐

Define that the network finally converged to x(k).

(23) $$x\left(k\right)=u\Gamma^{T}x\left(0\right)$$

where $u={\left[1,1,...,1\right]}^{T}$ and $\Gamma^{T}=\frac{w^{T}}{1+a^{k}\left(N-1\right)}\prod_{k=1}^{k_{0}-1}P\left(k\right)=\left[\Gamma_{1},\Gamma_{2},...,\Gamma_{N}\right]$. Since a>1, we derive that $\Gamma_{N}\geq ...\geq \Gamma_{2}\geq a\Gamma_{1}$ and $\Gamma_{1}+\Gamma_{2}+...+\Gamma_{N}=1$. Thus, $\Gamma_{1}\leq \frac{1}{N}$. Therefore, the weights of the misbehaving nodes can be reduced, but the misbehaving nodes are not eliminated by the proposed scheme. $\Gamma_{2}\geq a\Gamma_{1}$; the effect of the misbehaving nodes becomes smaller when *a* becomes larger. Additionally, our scheme is especially efficient when the false nodes attack the network continuously.

## 4. Evaluation

This section presents numerical examples to illustrate the PACS algorithm. We validate the efficiency of PACS by comparing the consensus results without attackers and with data falsification attackers. The algorithm presented by Olfati-Saber [13] is called the Olfati algorithm here. The proposed PACS and the Olfati algorithm are compared in this section.

### 4.1. Experiments Setup

We get the experiment reports at an apartment in Shanghai Jiao Tong University (SJTU). We use nine USRPs (Universal Software Radio Peripherals) with a broadband antenna (70–1000 MHz) and a TVRX daughterboard (50–860-MHz receiver) to detect three channels of TV broadcasts and three relay nodes to transmit information. The nodes are deployed in a 10 m × 10 m area. Energy detection is adopted here because of its short sensing time and simplicity. Although the positions of some nodes are very close, there are big differences among the sensing reports. Table 1 shows the sensing reports of close node pairs; (5, 6) and (8, 9) could be quite different. These features illustrate that it is hard to distinguish the diversity of sensing reports caused by data falsification or not, and it is impractical to judge malicious nodes only by using a threshold. Therefore, we design a consensus secure scheme to overcome the diversity of sensing data and the unsafe factors in the network. The consensus algorithm can solve the problem of the sensing data diversity. Additionally, the adjusted parameters in the consensus algorithm can reduce the effect of uncertainties in the network.

Throughout the numerical examples, the initial sensing value (*i.e.*, $x_{i}\left(0\right)$) is the average of 300 sensing reports for the band of 750–758 MHz. We set 10% as the initial link loss rate of each link, *i.e.*, $a_{ij}=0.90$. Then, we use the off-line data analysis to validate the efficiency of the proposed scheme. Weights for RNs are set to be $\gamma_{ij}=a_{ij}/\Sigma_{j\in N_{i}}a_{ij},\forall i\in I_{R}$. The algorithm is implemented with the decreasing weight sequence σ(k)=1/10, k≤20 and σ(k)=1/(k-1),k>20, and a=5.

### 4.2. Numerical Example 1

**Figure 1:**
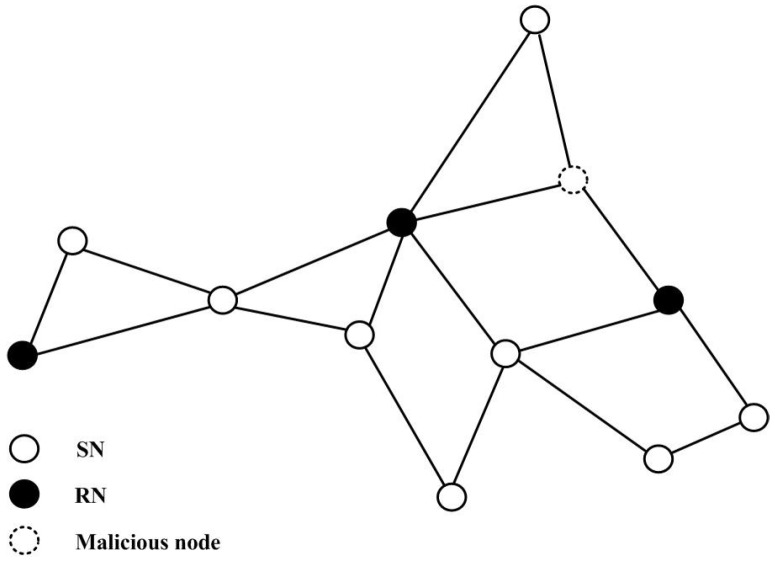
A network with eight honest sensor nodes, three relay nodes and one attacker.

**Figure 2:**
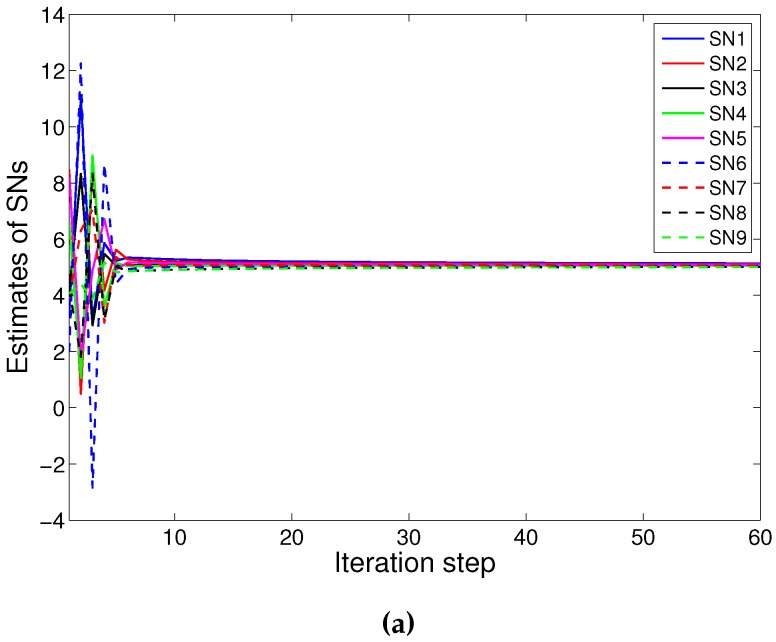

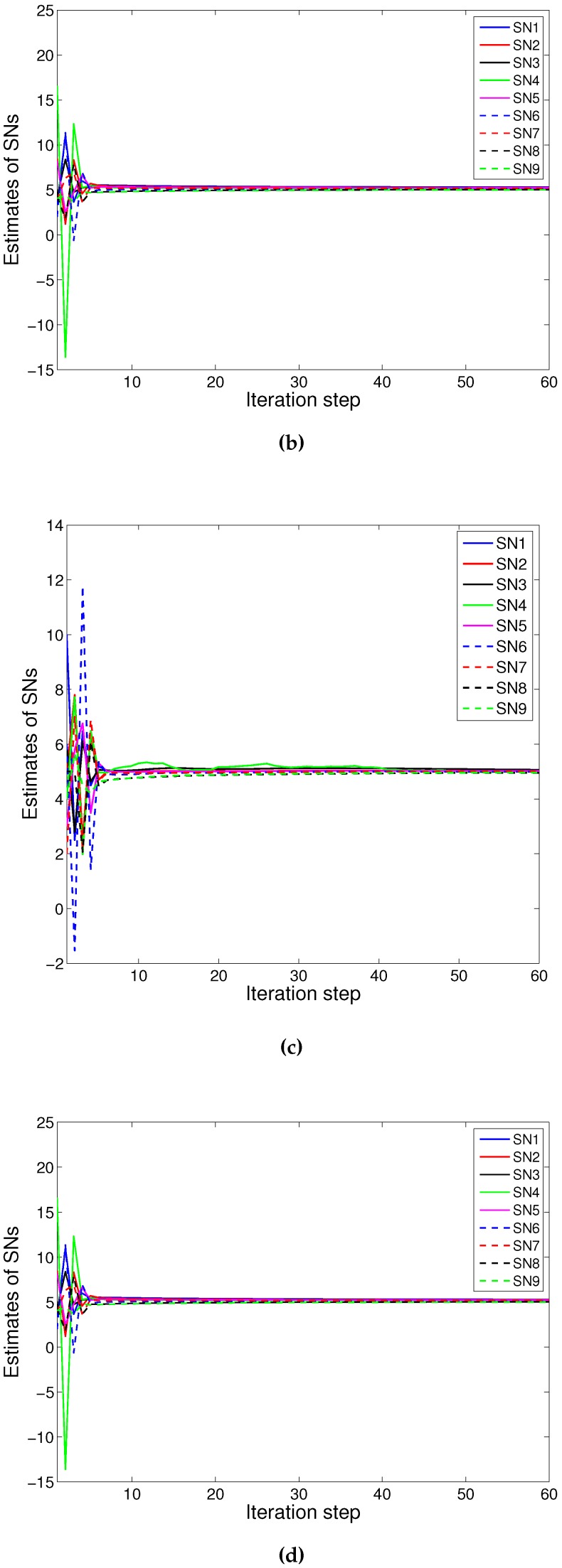
Convergence of parameter adjusted-based consensus (PACS): (**a**) without attackers; (**b**) with one *Perception Data Falsification (PDF)* attacker; (**c**) with one *Iteration Data Falsification (IDF)* attacker; (**d**) with one *Random Data Falsification (RDF)* attacker.

In this experiment, we select 12 sensors: nine SNs and three RNs; shown in Figure 1. Firstly, we illustrate the results with PACS. Figure 2a shows the result without attackers. The network converges to 4.9864. Then, we consider the network attacked by Node 4. The attacker executes the *PDF* attack and forges 16.5966 as the sensing value. Figure 2b shows the results, and the consensus value is 5.0013. Furthermore, Node 4 is set to broadcast the adjusting value with $x_{4}\left(k\right)=x_{4}\left(k\right)+\omega_{4}\left(k\right)$ where $\omega_{4}$ is randomly selected in [−0.5, 0.5] in the case of the *IDF* attack. The estimated value of the attacker may fluctuate in the data fusion process, but the network converge to 4.9543, as fast as in Figure 2c. Then we take the *RDF* attack into account; the attacker manipulates the sensing state value and adds Gaussian white noise to the states in each iteration step randomly. The result by PACS is demonstrated in Figure 2d. The network can converge to 5.0007 very quickly. We can see that the differences between the convergence value with attackers and without attackers are less than 0.1 (*i.e.*, the error is less than 2%). It is considered that the proposed scheme defends against the data falsification attack effectively.

**Figure 3:**
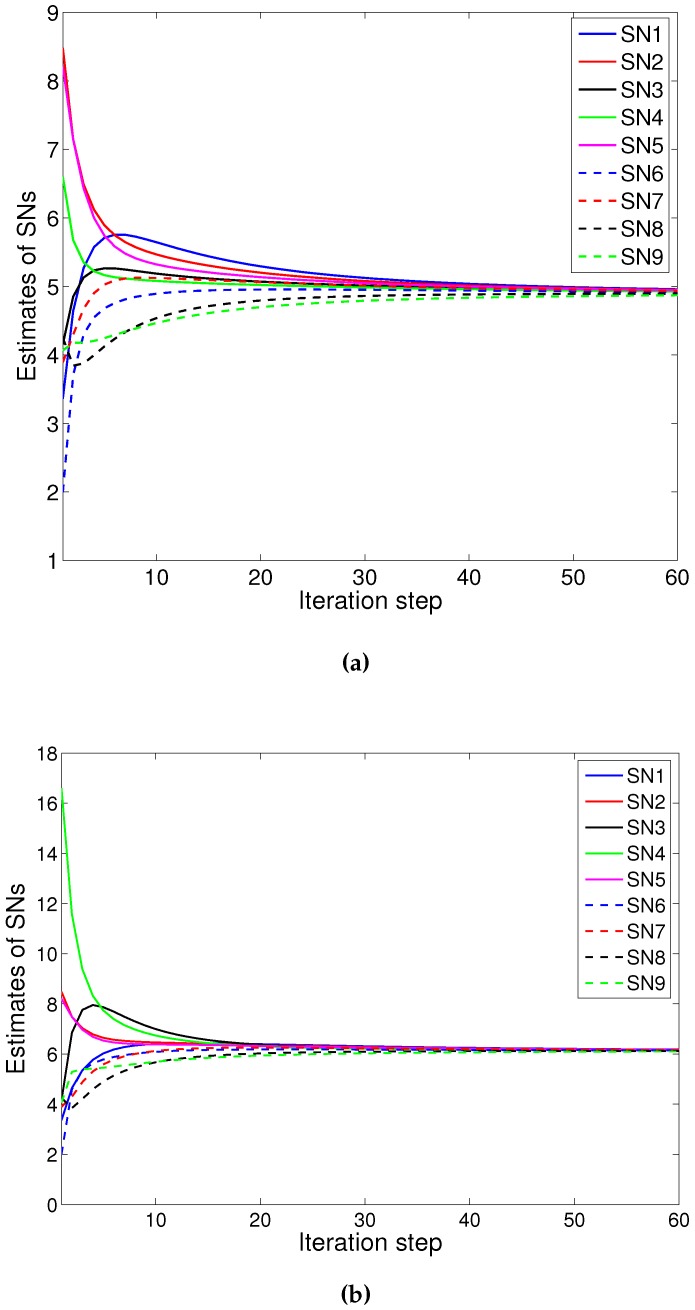

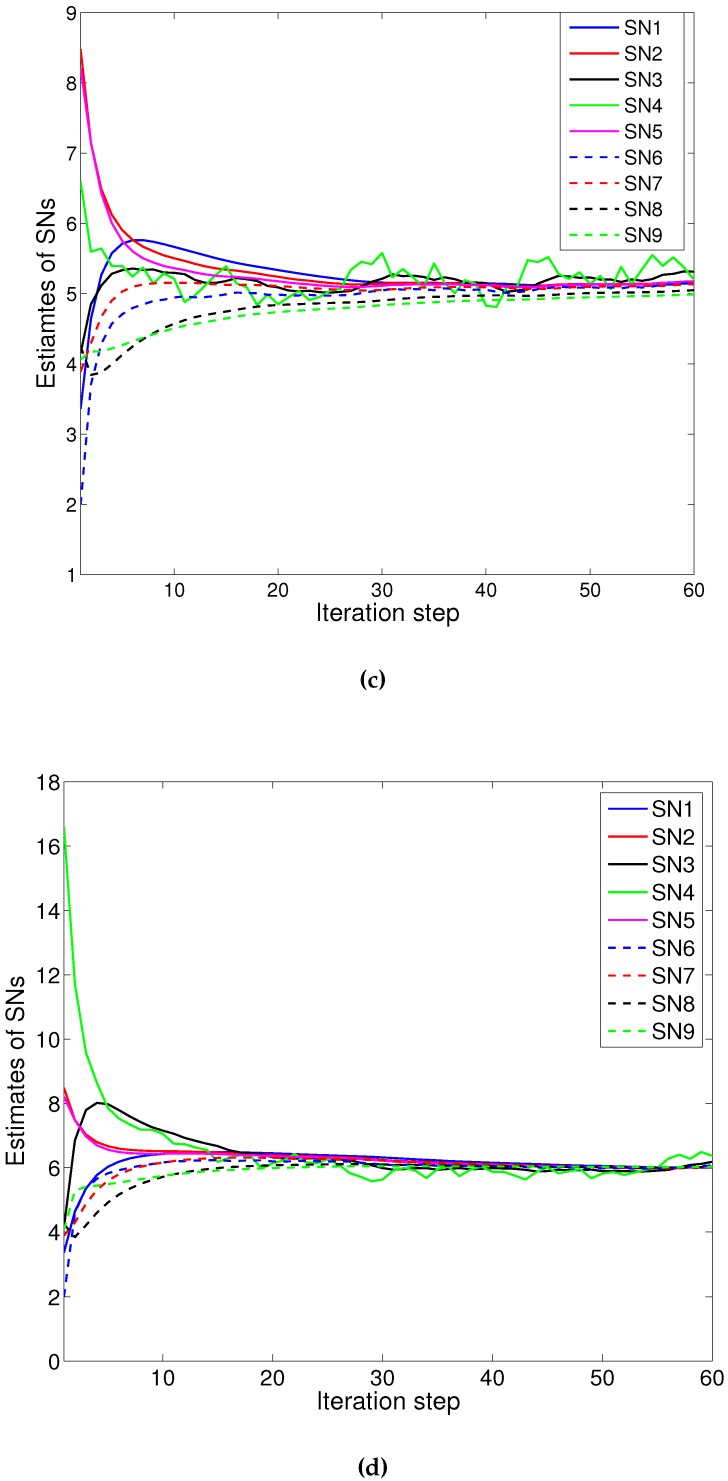
Convergence of the Olfati algorithm: (**a**) without attackers; (**b**) with one *SDF* attacker; (**c**) with one *IDF* attacker; (**d**) with one *RDF* attacker.

Then, considering the same attack with the above simulation, we show the simulation results with the Olfati algorithm. Figure 3a shows the result without attackers. The consensus results under three different types of attackers are shown in Figure 3b–d, respectively. We can see that the attacks make the network converge to a wrong value or even destroy the convergence of the network.

### 4.3. Numerical Example 2

**Figure 4:**
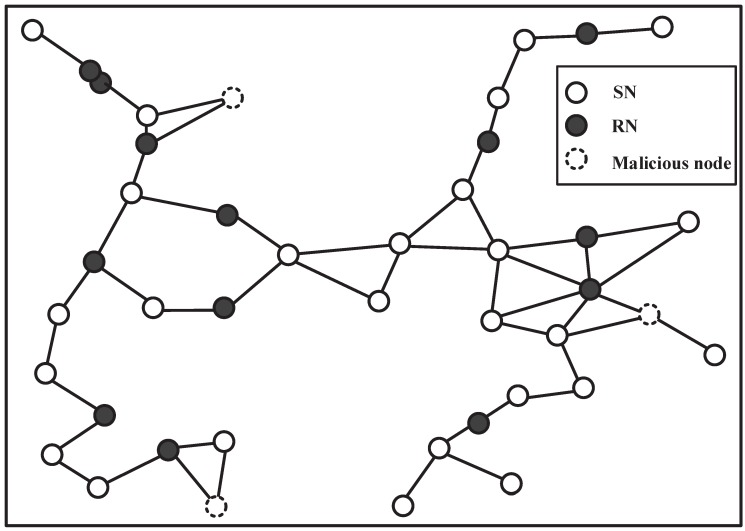
A network with 27 honest sensor nodes, 13 relay nodes and three attackers.

**Figure 5:**
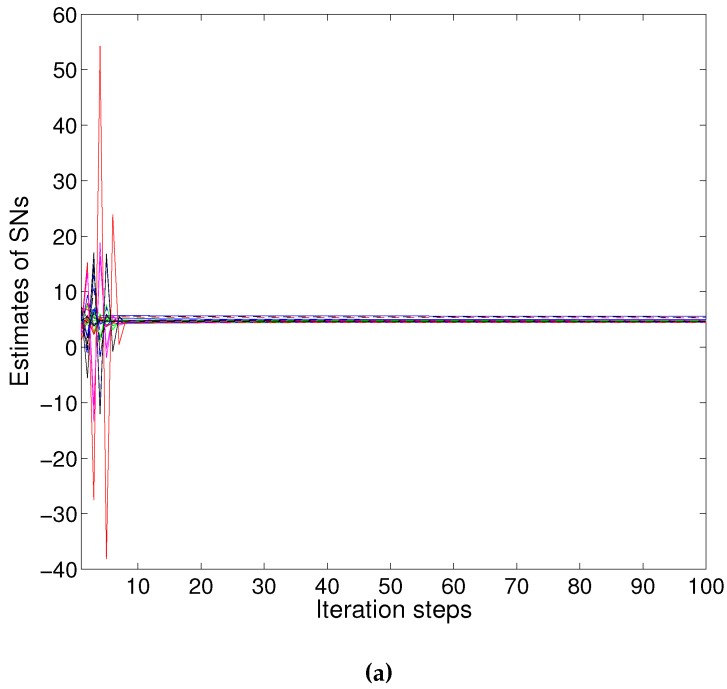

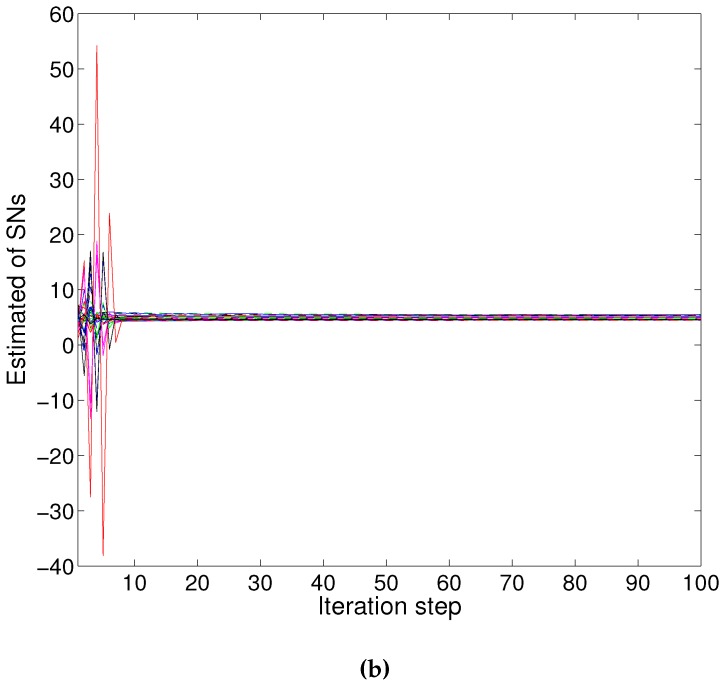
Convergence of PACS in numerical Example 2: (**a**) without attackers; (**b**) with three attackers.

We consider more sensors as in Figure 4. Firstly, the results of PACS are demonstrated. Figure 5a shows the results of the PACS algorithm on the network without attackers. The consensus result is 4.7543. Additionally, the convergence is quick, although there are more sensor nodes in the network. Then, we consider the case that there are three attackers executing three different attacks: the *SDF*, *ISF* and *RDF* attack, respectively. Figure 5b demonstrates that the value of $x_{i}\left(k\right)$ converges to 4.8561, and the difference between this value with the result without the attack is 0.1018 (*i.e.*, the error is less than 2.14%). We can see that the scheme has a quite good resistance against the variety of data falsification attacks that exists in the network simultaneously.

**Figure 6:**
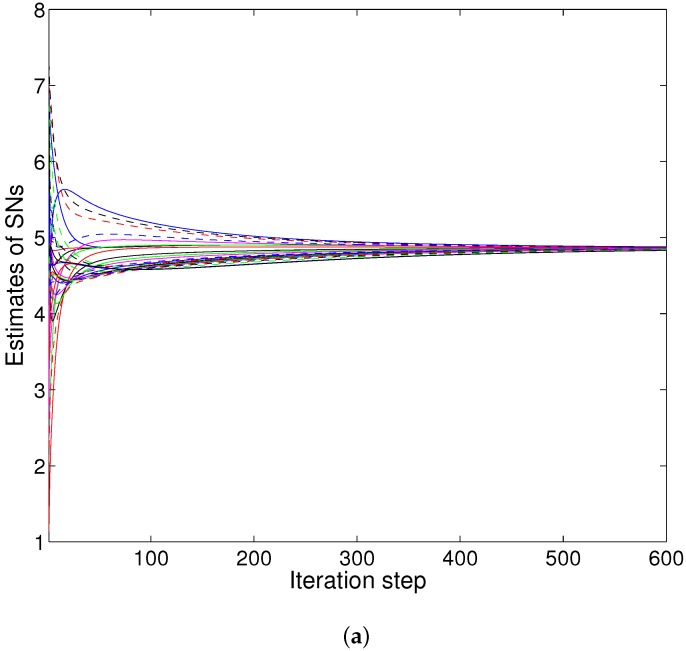

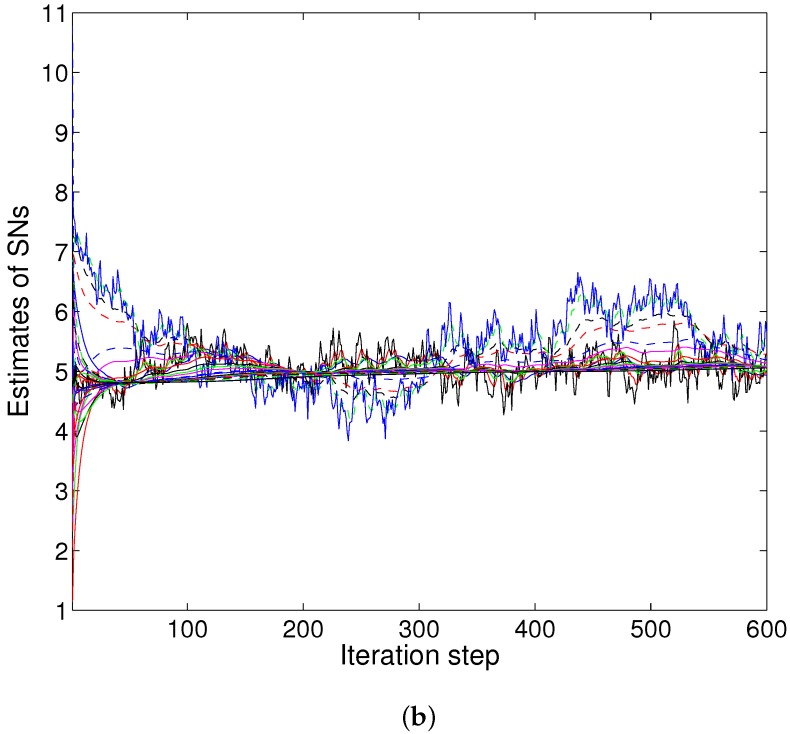
Convergence of Olfati in numerical example 2: (**a**) without attackers; (**b**) with three attackers.

Then, the results of Olfati algorithm on the network without attackers is shown in Figure 6a. The consensus speed is small, because the scale of the network is large. The consensus result is 4.7617. The same attackers are considered to execute the same types of attacks as the above. The results of the Olfati algorithm are shown as Figure 6b; the network cannot reach a consensus obviously.

## 5. Conclusions and Future Work

In this paper, we propose a secure scheme to reduce the destructive impact of the abnormal nodes in HWSNs. We utilize an undirected graph to represent the HWSNs and then introduce three different kinds of data falsification. A distributed detection algorithm with a local threshold is presented for classifying malicious nodes from honest ones. PACS is proposed to protect the network from the malicious nodes by decreasing their weights in the distributed estimation. The convergence property of PACS is proven to be guaranteed, and the simulation results illustrate the effectiveness and efficiency of the proposed scheme. We will study the issues of the attack under random graph topologies in heterogeneous wireless sensor networks in future work.

## Figures and Tables

**Table 1:** The sensing reports of sensor nodes (5, 6, 8, 9).

Region	662–670 MHZ	750–758 MHZ	798–806 MHZ
5	3.3626	8.4791	4.1553
6	6.5966	1.9973	8.2043
8	3.8923	4.2489	5.0492
9	2.8713	8.7158	3.9781
